# The mediating role of neuroticism and anxiety sensitivity in the relationship between childhood trauma and panic disorder severity

**DOI:** 10.3389/fpsyt.2024.1441664

**Published:** 2024-10-14

**Authors:** Fang He, Xiangyun Yang, Jia Luo, Pengchong Wang, Lijuan Yang, Zhanjiang Li

**Affiliations:** ^1^ Beijing Key Laboratory of Mental Disorders, National Clinical Research Centre for Mental Disorders, Beijing Anding Hospital, Capital Medical University, Beijing, China; ^2^ Advanced Innovation Center for Human Brain Protection, Capital Medical University, Beijing, China

**Keywords:** childhood trauma, panic disorder, neuroticism, anxiety sensitivity, mediating effect

## Abstract

**Objective:**

Despite advancements in understanding panic disorder (PD), its etiology and pathogenesis remain elusive, particularly concerning the influence of psychosocial factors. This study aims to explore the mediating roles of neuroticism and anxiety sensitivity (AS) in the relationship between childhood trauma and PD severity.

**Methods:**

We conducted a cross-sectional analysis involving 84 individuals diagnosed with PD and 112 age- and gender-matched healthy controls (HC). Childhood trauma was assessed using the validated Childhood Trauma Questionnaire (CTQ), while anxiety sensitivity levels were measured using the Anxiety Sensitivity Index-3 (ASI-3). Neuroticism was evaluated using the Chinese Big Five Personality Inventory (CBF-PI-B). The severity of PD was determined using the Panic Disorder Severity Scale (PDSS). Statistical analysis encompassed both correlation and mediation techniques to thoroughly examine the relationships among childhood trauma, neuroticism, AS, and PD severity.

**Results:**

Individuals with PD exhibited significantly higher CTQ, ASI-3, and neuroticism scores compared to HC (all P<0.05). Within the PD group, strong positive correlations were found between CTQ scores, ASI-3 scores, neuroticism levels, and PDSS scores, with correlation coefficients ranging from 0.336 to 0.504 (all P<0.001). Additionally, our results indicated that neuroticism and AS acted as mediating factors in the relationship between childhood trauma and the severity of PD, with the 95% confidence interval for the indirect effects ranging from 0.028 to 0.116.

**Conclusion:**

This study highlights that PD patients exhibit significantly higher levels of childhood trauma, neuroticism, and AS compared to HC. Furthermore, childhood trauma indirectly influences PD severity through a chain mediation involving neuroticism and AS. These findings suggest the importance of psychological factors in moderating the impact of early adverse experiences on the development and progression of PD.

## 1 Introduction

Panic disorder (PD) is characterized by recurrent and abrupt episodes of intense panic and unpredictable anxiety, accompanied by symptoms of autonomic hyperactivity such as palpitations, shortness of breath, sweating, and tremors. Patients may also experience a sensation of asphyxia, impending doom, and loss of control. PD significantly impacts patients’ quality of life and social function, imposing a substantial burden on both families and society as a whole (1). According to findings from the 2019 China Mental Health Survey, the annual prevalence rate of PD in China is 0.3%, with a lifetime prevalence rate of 0.5% (2). Despite ongoing research efforts, the etiology and mechanisms underlying PD remain poorly understood. In addition to biological factors, numerous studies have identified psychological factors such as childhood trauma, neuroticism, and anxiety sensitivity as contributors to both the onset and severity of PD (3, 4).

Childhood trauma refers to the traumatic events that an individual encounters during childhood and adolescence, involving physical, psychological, and sexual damage (5). It serves as a significant stressor that has been shown to increase an individual’s susceptibility to PD and is a risk factor for its development and progression. Moreover, the severity of PD has been consistently linked to childhood trauma (4). Previous research indicates that individuals with a history of childhood trauma tend to experience more frequent and severe PD episodes than those without such experiences (6). Additionally, a recent study suggests that childhood trauma may influence the development of personality traits, particularly elevating the levels of neuroticism (7). Neuroticism plays a pivotal role in the development of PD, as individuals with higher neuroticism levels tend to exhibit greater anxiety under stress. Consequently, neuroticism may act as a mediator between childhood trauma and PD severity (8).

The cognitive model theory of PD, proposed by Beck and Clark, posits that PD episodes arise from fear and a catastrophized interpretation of anxiety-related physical sensations (9). This cognitive-behavioral perspective views PD as a manifestation of a ‘fear of fear’. Reiss and McNally introduced the concept of Anxiety Sensitivity (AS) to elucidate this phenomenon. AS refers to an individual’s apprehension of anxiety-related sensations, which can exacerbate anxiety and fear, ultimately precipitating PD episodes (10). This fear arises from m the belief that these symptoms will have detrimental effects on physical, cognitive, and social functioning and represent a relatively stable cognitive trait (11). Numerous studies have demonstrated that AS not only predisposes individuals to PD but also correlates positively with the severity of PD symptoms (3, 11).

The Big Five personality structure theory suggests that neuroticism reflects individual differences in emotional stability and affects emotional response patterns. Individuals with high neuroticism levels tend to experience more anxiety, worry, and other negative emotions, and are particularly sensitive to negative emotional stimuli. Several studies have indicated a significant positive correlation between neuroticism and AS (12), and neurotic individuals may exacerbate the severity of PD by increasing AS (13).

The relationship between neuroticism and AS is a topic of extensive discussion. Neuroticism, a personality trait, refers to an individual’s inclination toward emotional instability and negative emotional responses under stress (14). AS, on the other hand, pertains to an individual’s fear and concern regarding anxiety symptoms such as heart palpitations and sweating (15). This fear is not rooted in anxiety itself but rather in the apprehension of potential negative consequences of these symptoms, such as a heart attack. Although both neuroticism and AS exhibit overlap in their response to negative emotions, they represent distinct concepts. Neuroticism encompasses a broad personality trait, while AS denotes a specific psychological response to anxiety symptoms. Research has indicated that childhood trauma significantly influences an individual’s mental health and is closely associated with the development of both neuroticism and AS. Experiences of childhood trauma, including abuse, neglect, or other adverse events, may elevate neuroticism levels and increase sensitivity to anxiety symptoms. Emotional reactivity and negativity can exacerbate neuroticism, while overinterpretation and fear of physical symptoms may increase AS (16). Understanding the interplay between neuroticism, AS, and childhood trauma can enhance our comprehension of their impact on mental health and inform the development of psychological interventions. However, despite growing recognition of the intertwined nature of socio-psychological constructs such as childhood trauma, neuroticism, and AS with the increased risk of PD, the specific psycho-pathological pathways linking these factors to the disorder’s severity, particularly within the context of childhood trauma, remain relatively unexplored.

Building upon existing evidence, this study aims to investigate the mediating roles of neuroticism and AS in exacerbating childhood trauma and the severity of PD. We hypothesize that neuroticism and AS mediate the relationship between childhood trauma and PD severity, with a potential sequential mediation. Adopting a cross-sectional study design, we will assess these variables in patients diagnosed with PD and healthy controls (HC). The research will provide empirical support for proactive and therapeutic interventions in the management of PD.

## 2 Materials and methods

### 2.1 Participants

A cross-sectional study was conducted on outpatients diagnosed with PD at Beijing Anding Hospital from April 2023 to December 2023 using convenient sampling. The inclusion criteria were as follows: (1) individuals who met the DSM-5 diagnostic criteria for PD, (2) had a total Panic Disorder Severity Scale (PDSS) score ≥ 7, (3) were aged between 18 and 60, (4) had no gender restrictions, and (5) possessed an education level of at least middle school. Exclusion criteria included: (1) patients with organic brain diseases and major physical illness, (2) diagnosis of schizophrenia or bipolar disorder, and (3) drug dependence or psychoactive substance abuse. The sample size was determined using a power analysis conducted with G*Power (version 3.1). The analysis aimed to detect medium effect sizes (Cohen’s d = 0.5) with a statistical power of 80% (1 - β = 0.80) at a significance level of α = 0.05. Based on the results of the power analysis, a minimum of 80 participants was required to achieve adequate statistical power. To account for potential attrition or incomplete responses, the final sample size was slightly increased beyond this requirement. Initially, 90 PD patients were selected, but after excluding six cases due to invalid questionnaires, a total of 84 patients (93.3%) completed all assessments with valid responses. Among them, 44 were females and 40 were males, with a mean age of 33 ± 9 years, a mean education level of 15.0 ± 2.6 years, and disease duration ranging from 0.8 to 2.5 years.

Concurrently, HC matching PD patients in terms of age, gender, and education level were recruited from the community. The inclusion criteria for HC were as follows: (1) age between 18 and 60 years, (2) no gender restrictions, (3) an education level of junior high school or higher, and (4) a PDSS total score of less than 7 points. Exclusion criteria were similar to those of the PD group. The study included a total of 112 healthy volunteers, comprising 59 females and 53 males, with an average age of 34 ± 7 years and an average education level of 15.2 ± 2.7 years. No statistically significant differences were observed between the control and PD groups in terms of gender, age, and education level. All participants volunteered for the study and provided informed consent.

The research protocol was approved by the Research Ethics Committee of Beijing Anding Hospital, Capital Medical University, Beijing, China [(2023) Scientific Research No. (60)-202395FS-2].

### 2.2 Measures

#### 2.2.1 General demographic information

The research group administered the General Information Questionnaire, which gathered data on subjects’ gender, age, nationality, education level, marital status, occupation, age of onset, disease course, family history, and prior treatments.

#### 2.2.2 Childhood trauma questionnaire

The CTQ, developed by Bernstein’s team in 2003, comprises 28 items rated on a 5-point scale. It assesses five dimensions: emotional abuse, physical abuse, sexual abuse, emotional neglect, and physical neglect (17). The degree of childhood trauma is directly proportional to the score obtained. A higher score indicates a greater degree of childhood trauma. The Chinese version, translated and revised by Zhao Xingfu et al., has demonstrated strong validity and reliability (18).

#### 2.2.3 Chinese big five personality inventory brief version

The questionnaire consists of 40 items, categorized into five subscales: neuroticism, extroversion, agreeableness, conscientiousness, and openness. Each subscale was scored on a 6-point scale ranging from 1 (not at all) to 6 (completely), with higher scores indicating more vital personality traits. For the current study, only the neuroticism subscale was used, with higher scores indicating greater levels of neuroticism. The neuroticism subscale of the CBF-PI-B has demonstrated good internal consistency, with a Cronbach’s alpha coefficient of 0.814 (19). Test-retest reliability over a 10-week period was reported to be 0.811, indicating strong temporal stability. Factor analysis has confirmed the validity of the neuroticism subscale as part of the broader CBF-PI-B, with all items loading above 0.40 on the intended factor.

#### 2.2.4 Anxiety sensitivity index-3

The ASI-3 was used to evaluate AS and fear of anxiety-related symptoms (20). It comprises 18 items distributed across three subscales: physical concern, social concern, and cognitive concern. Each subclass contains six items, and each item is scored on a 5-point scale ranging from 0 (minor) to 4 (a lot). The scale has been validated for use in various populations, including college students and patients with anxiety disorders. The Chinese version of ASI-3 has been applied in clinical research, demonstrating robust reliability and validity, with a reported Cronbach’s α coefficient of 0.902 (21).

#### 2.2.5 Panic disorder severity scale

The PDSS was used to assess the severity of PD, monitor changes in symptoms, and evaluate treatment effectiveness. It consists of seven items, assessing the impact of panic attacks on different aspects of life, including frequency, pain, anticipatory anxiety, place avoidance, physical sensations, social function, and occupational function. Each item is scored on a 5-point scale (ranging from 0 to 4, indicating none, mild, moderate, severe, or extremely severe). The total score ranges from 0 to 28, with interpretations as follows: scores of 0-1 are considered normal, 2-5 indicate borderline severity, 6-9 suggest mild severity, 10-13 indicate moderate severity, 14-16 signify severe severity, and scores >17 indicate extreme severity. The scale demonstrates good validity and reliability, with a consistent reliability coefficient of 0.795.

### 2.3 Evaluation methods

PD patients were diagnosed by two deputy senior psychiatrists at Beijing Anding Hospital. PDSS examinations for both groups were conducted by four clinical psychologists in a well-lit and quiet room, providing unified guidance. Participants completed demographic questionnaires, CTQ, CBF-PI-B, and ASI-3 self-rating scales. Questionnaires with a vacancy rate or incompleteness exceeding 20% were deemed invalid. Subsequently, general information and clinical data were collected. Quality control measures were implemented before the study, including training on the technical route and all assessment scales for consistency. The researchers’ assessment consistency test of PDSS achieved a score of more than 0.80.

### 2.4 Statistical analysis

Statistical analyses were performed using SPSS software, version 27.0. Descriptive statistics for categorical variables were reported as frequencies and percentages, while continuous variables adhering to a normal distribution were expressed as means ± standard deviations (SD). Comparative analysis between the PD and control groups regarding age, ASI-3 scores, levels of neuroticism, experiences of childhood trauma, and PDSS scores was conducted using independent samples t-tests. For variables not following a normal distribution, medians and interquartile ranges (IQR) were reported. Pearson correlation analysis was applied to explore the associations among childhood trauma, neuroticism, AS, and PDSS scores within the PD group. All statistical tests were set at an alpha threshold of 0.05. To examine the mediating roles of neuroticism and AS in the relationship between childhood trauma and PDSS scores, this study employed Model 6 from Hayes’ PROCESS analysis for serial mediation testing. The proposed model designated childhood trauma as the independent variable, PDSS as the dependent variable, and neuroticism and AS as mediator variables. The significance of the serial mediation effects was tested using a bias-corrected bootstrap approach with 5,000 resamples to obtain 95% confidence intervals (CIs) for the parameter estimates.

## 3 Results

### 3.1 Comparison of demographic data, ASI-3, neuroticism, childhood trauma and PDSS between PD and HC groups

In **Table 1**, the comparison of demographic and clinical characteristics between the PD and HC groups shows no significant differences in gender distribution and median age, indicating demographic homogeneity across the groups. Educational attainment, categorized as junior high school, high school, junior college, and bachelor’s degree or above, demonstrated no statistically significant variances between the two groups (*p*-values: junior high school = 0.933, high school = 1.000, junior college = 1.000, and bachelor’s degree or above = 0.902), suggesting comparable levels of education.

**Table 1:** Comparison of sociodemographic characteristics and psychological scale scores between patients with Panic Disorder (PD) and Healthy Controls (HC).

Variables	PD (n=84)	HC (n=112)	*P-*value
Sex = male (%)	34 (40.5)	39 (34.8)	0.509
Age	32.00 [26.75, 39.00]	30.00 [27.00, 40.00]	0.817
Junior high school	14	17	0.932
High school	22	30	0.918
Junior college	30	39	0.914
Bachelor’s degree or above	18	26	0.902
ASI-3***	32.00 [20.00, 44.50]	12.00 [3.50, 25.00]	<0.001
ASI-P***	13.00 [9.00, 17.00]	3.00 [0.00, 8.00]	<0.001
ASI-S***	11.00 [7.00, 15.25]	6.00 [2.75, 10.00]	<0.001
ASI-C***	8.00 [4.00, 12.25]	3.00 [0.75, 7.00]	<0.001
Neuroticism*	29.24 (7.43)	22.12 (7.29)	<0.001
Childhood trauma*	46.00 [41.00, 52.25]	42.00 [37.00, 48.00]	<0.001

ASI-3, Anxiety Sensitivity Index-3; ASI-P, Anxiety Sensitivity Index-physical; ASI-S, Anxiety Sensitivity Index-social; ASI-C, Anxiety Sensitivity Index-cognitive. ***P<0.001, *P<0.05.

Significant distinctions emerged in the ASI-3 subscale scores, with the PD group exhibiting higher median scores in physical concerns (*p*<0.001), social concerns (*p*<0.001), and cognitive concerns (*p*<0.001), indicating the elevated AS levels among PD patients. Furthermore, the ASI-3 total score was significantly higher in the PD group (32.00 [IQR: 20.00-44.50]) compared to the HC group (12.00 [IQR: 3.50-25.00], *p*<0.001), indicating overall elevated anxiety sensitivity levels among PD patients.

Additionally, the PD group reported significantly higher mean neuroticism scores (*p*<0.001) and greater childhood trauma (*p*<0.001), further emphasizing the psychological and experiential differences between the PD and HC groups.

### 3.2 Correlation analysis between childhood trauma, neuroticism, and AS in PD patients

In assessing the interrelations among childhood trauma, neuroticism, AS, and PD severity within the PD cohort, several statistically significant correlations were observed. We performed a correlation analysis to examine the relationships between childhood trauma, neuroticism, anxiety sensitivity, and panic disorder severity. The statistical methods applied to compute these correlations are detailed in the Methods section, and the results are summarized in **Table 2**. Childhood trauma exhibited moderate correlations with neuroticism (r=0.381, *p*<0.01) and AS (r=0.532, *p*<0.01), and a mild correlation with PD severity (r=0.336, *p*<0.01). Neuroticism displayed a strong correlation with AS (r=0.591, *p*<0.01) and a moderate correlation with PD severity (r=0.473, *p*<0.01). AS was strongly correlated with PD severity (r=0.504, *p*<0.01). These correlations highlight the complex interplay between childhood experiences, personality traits, and the severity of PD symptoms, emphasizing the multifaceted nature of PD etiology.

**Table 2:** Correlation analysis between childhood trauma, neuroticism, AS, and PD severity.

Variables	*Mean ± SD*	1	2	3	4
Childhood trauma	50.82 +/- 13.78				
Neuroticism	29.24 +/- 7.43	0.381 **			
Anxiety sensitivity	30.90 +/- 12.97	0.532 **	0.591 **		
Panic disorder severity	12.49 +/- 2.94	0.336 **	0.473 **	0.504 **	

**P<0.01.

### 3.3 Chain mediation analysis of neuroticism and AS

This study employed childhood trauma as the independent variable, with neuroticism and AS as mediating variables, and the severity of PD as the dependent variable. Multivariate hierarchical regression analysis was performed using model 6 in the PROCESS plug-in within SPSS software, following the guidelines outlined in Hayes’s SPSS macro program manual.

**Figure 1:**
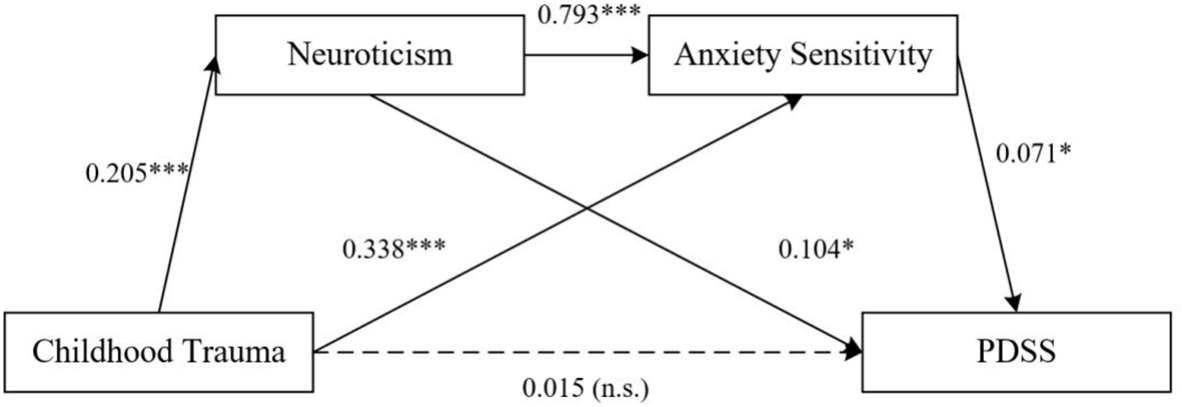
Mediating Effects of Neuroticism and Anxiety Sensitivity on the Relationship Between Childhood Trauma and PD Severity. *P<0.05, ***P<0.001. n.s., not significant.

As depicted in **Table 3**, childhood trauma significantly predicted the severity of PD (*β=0.072, t=3.229*, p<0.01), neuroticism (*β*=0.205, *t*=3.728, *p*<0.001), and AS (*β*=0.338, *t*=4.063, *p*<0.001). Neuroticism positively predicted AS (*β*=0.793, *t*=5.141, *p*<0.001) and the severity of PD (*β*=0.104, *t*=2.257, *p*<0.05), while AS significantly predicted the severity of PD (*β*=0.071, *t*=2.454, *p*<0.05). However, when neuroticism and AS were included in the regression equation, childhood trauma did not significantly predict the severity of PD (*β*=0.015, *t*=0.637, *p*>0.05). The path coefficients and significance are displayed in **Figure 1**.

**Table 3:** Regression analysis of neuroticism and AS in the chain model between childhood trauma and PD severity.

Variables	Neuroticism			Anxiety sensitivity			Panic disorder severity		
	*β*	*SE*	*t*	*β*	*SE*	*t*	*β*	*SE*	*t*
Childhood trauma	0.205	0.055	3.728 ***	0.338	0.083	4.063 ***	0.015	0.024	0.637
Neuroticism				0.793	0.154	5.141 ***	0.104	0.046	2.257 *
Anxiety sensitivity							0.071	0.029	2.454 *
*R²*	0.145			0.459			0.304		
*F*	13.901 ***			34.408 ***			11.670 ***		
*p*	0.000			0.000			0.000		

*P<0.05, ***P<0.001.

The study employed the non-parametric percentile Bootstrap method, as suggested by Hayes, to assess the mediation effect. The findings revealed that the direct effect of childhood trauma on PD severity was not significant (estimates = 0.015, 95% CI [-0.023, 0.053]). However, the total indirect effect of neuroticism and AS was 0.057, with the Bootstrap 95% CI not including a value of 0, indicating a significant mediating effect of these variables between childhood trauma and PD severity. These results suggest that neuroticism and AS act as chain mediators between childhood trauma and the severity of PD.

The specific mediating effect showed three paths: (1) the indirect effect from childhood trauma to neuroticism to the severity of PD, the effect size was 0.021; (2) the indirect effect from childhood trauma → AS → severity of PD, the effect size was 0.024; (3) the indirect effect from childhood trauma → neuroticism → AS → severity of PD, the effect size was 0.012. The confidence intervals of indirect effect (1), indirect effect (2), and indirect effect (3) did not include a 0 value, indicating that the indirect effect produced by this pathway reached a statistically significant level (**Table 4**). These results highlight the complex interplay between childhood trauma, neuroticism, AS, and PD severity, demonstrating the significant chain mediation effect of neuroticism and AS on the impact of childhood trauma on PD symptoms.

**Table 4:** Mediating roles of neuroticism and AS on the relationship between childhood trauma and PD severity.

Effects	Path	Estimates	Effect size	95% confidence interval	
				Lower limit	Upper limit
Direct effects	Childhood trauma → Panic disorder severity	0.015	20.95%	0.032	0.062
Indirect effect	Childhood trauma → Neuroticism → panic disorder severity	0.021	29.75%	0.004	0.045
	Childhood trauma → Anxiety sensitivity → Panic disorder severity	0.024	33.24%	0.002	0.048
	0.048 Childhood trauma → Neuroticism → Anxiety sensitivity → panic disorder severity	0.012	16.06%	0.001	0.030
Total indirect effect		0.057	79.05%	0.028	0.088
Total effect		0.072	100.00%	0.028	0.116

## 4 Discussion

In this study, we observed higher scores for ASI-3, neuroticism, childhood trauma, and PDSS in the PD group compared to the HC group, consistent with previous findings. PD patients exhibited elevated levels of AS compared to healthy individuals, along with a significantly higher prevalence of childhood trauma and a notable inclination towards neuroticism. These findings align with previous research, supporting the hypothesis that neurotic personality traits and AS mediate the relationship between childhood trauma and PD severity (3, 4). Our study also reveals that neuroticism and AS form a complete mediation pathway between childhood trauma and PD severity. As shown in **Figure 1** and **Table 4**, our chain mediation model reveals that childhood trauma exerts its effects on PD severity through a dual pathway involving both neuroticism and AS. The first pathway involves the direct influence of childhood trauma on neuroticism, which then increases AS (β = 0.793, p < 0.001). Neuroticism acts as a catalyst, exacerbating the cognitive predisposition to fear anxiety-related symptoms, thus reinforcing the “fear of fear” cycle that is characteristic of PD. The second pathway highlights the direct relationship between childhood trauma and AS (β = 0.338, p < 0.001), followed by AS’s significant impact on PD severity (β = 0.071, p < 0.05). Together, these pathways explain how early adverse experiences contribute to the development and maintenance of PD through intertwined psychological mechanisms.

Neurotic personality traits act as a fundamental factor for the onset of PD, while AS represents a cognitive vulnerability that predisposes individuals to intensely fearful reactions towards anxiety symptoms. Neuroticism can amplify AS (13), and childhood trauma-induced physiological arousal exacerbates the perception of anxiety-related somatic responses as threats, perpetuating a cycle of “fear of fear.” This cycle enhances AS, linking physical discomfort to intense fear, thus perpetuating panic attacks and worsening PD symptoms.

Childhood trauma indirectly impacts PD severity through the mediation of neuroticism and AS, as demonstrated in our model (**Figure 1**). Theoretical frameworks propose that childhood trauma heightens environmental vigilance, causing individuals to misinterpret ambiguous stimuli as imminent threats. This increased vigilance, coupled with maladaptive cognitive strategies such as self-blame, rumination, and catastrophizing, contributes to the development of neurotic personality traits characterized by heightened nervousness, fear, and anxiety (8). Neurobiological research indicates that childhood trauma triggers the activation of the hypothalamic-pituitary-adrenal axis, excessive excitation of the sympathetic nervous system, and increased production of catecholamines, leading to physiological reactions such as elevated heart rate and breathlessness (22). Additionally, childhood trauma can alter brain structures, including the hippocampus, amygdala, and cerebellum, resulting in heightened aggression, disinhibitory disorders, and increased vulnerability to stress (23). These neurobiological changes contribute to the development of AS and neuroticism, which in turn exacerbate PD symptoms.

Childhood trauma also disrupts cognitive function and alters the perception of external stimuli (24). This disruption leads to catastrophic thinking patterns, increased cognitive attention to AS, and heightened fear associated with catastrophic thoughts, which are cognitive mechanisms underlying PD. Consequently, PD symptoms persist and intensify. Moreover, individuals who experience childhood trauma often exhibit low self-esteem and struggle with trust and interpersonal relationships (25). These experiences foster anxiety and avoidance in social interactions, leading to increased AS and impairments in social functioning (26). Addressing these aspects in therapeutic interventions could be crucial for improving outcomes in PD patients.

Clinically, these findings underscore the need for tailored psychotherapeutic interventions that address the impact of childhood trauma on neuroticism and AS. Interventions that focus on reducing maladaptive cognitive strategies and managing AS could potentially mitigate the impact of childhood trauma on PD severity, thereby improving treatment outcomes for patients with a history of childhood trauma.

### 4.1 Limitations

However, this study has several limitations that warrant consideration. First, the retrospective assessment of childhood trauma may introduce recall bias, potentially affecting the accuracy of reported experiences. Second, the cross-sectional design prevents establishing causal relationships between PDSS risk factors and their correlates, necessitating cautious interpretation. Additionally, the study did not differentiate between PD patients with and without agoraphobia at the time of diagnosis. This lack of differentiation could influence the interpretation of PD severity and its associated factors. Future research should address these limitations by employing longitudinal designs to capture causal inferences and temporal relationships post-childhood trauma, and by distinguishing between PD subtypes to enhance the specificity and applicability of the findings.

## 5 Conclusion

In conclusion, our study significantly advances our understanding of how childhood trauma influences PD severity, highlighting the mediating roles of neuroticism and AS. It underscores that individuals with childhood trauma history may exhibit higher neuroticism and AS levels, exacerbating PD symptoms. This model elucidates the psychological pathways linking early trauma to PD and emphasizes the importance of considering personality traits and cognitive sensitivities in therapeutic interventions. By identifying and targeting these mediating factors, interventions can be tailored to effectively mitigate PD symptom severity, enhancing overall quality of life and mental health for those affected.

## Data Availability

The datasets presented in this article are not readily available due to concerns regarding the privacy and confidentiality of participant information. Requests to access the datasets should be directed to water1904@163.com.
